# Mathematically Directed Single-Cut Osteotomy

**DOI:** 10.3390/medicina58070971

**Published:** 2022-07-21

**Authors:** Stephen J. Wallace, Joseph T. Patterson, Sean E. Nork

**Affiliations:** 1 Summit Orthopaedics, Lake Oswego, OR 97035, USA; 2 Department of Orthopaedic Surgery, Keck School of Medicine of the University of Southern California, Los Angeles, CA 90033, USA; josephp7@usc.edu; 3 Harborview Medical Center, University of Washington, Seattle, WA 98104, USA; nork@uw.edu

**Keywords:** mathematically directed single-cut osteotomy, malunion, deformity

## Abstract

A mathematically directed osteotomy (MDO) is a surgical planning technique for correcting long bone deformities. Using a mathematically derived osteotomy plane, the single-cut correction simultaneously addresses angular deformity, axial malrotation, and minor shortening. This review describes an MDO’s indications for use, defines its input and output variables, includes the required graphs for osteotomy planning, and provides intraoperative tips and tricks for successful execution. Finally, the authors present a digital MDO calculator to simplify the complex computations and allow for more precise planning.

## 1. Introduction

A mathematically directed osteotomy (MDO) is an elegant surgical technique for correcting specific complex long bone deformities. Initially described for clinical use by brothers Bruce and Brian Sangeorzan in 1989, the method allows for simultaneous angular and torsional correction through a single osteotomy plane [1,2]. Only one cut is required, no bone wedge is removed, and the resultant bone surfaces are firmly opposed, permitting compressive internal fixation and direct bone healing across the osteotomy plane. In contrast to the oblique plane osteotomy [3], the mathematically directed single-cut osteotomy is precisely calculated to provide exact correction of an angular deformity in the sagittal and coronal planes while accounting for a consequential change in axial rotation to address a torsional deformity.

As originally described, the technique has four steps: (1) define the deformity, (2) determine the osteotomy angle required for the deformity correction, (3) establish the osteotomy starting point relative to the axial plane, and (4) rotate the bone into the corrected position for internal fixation.

This article reviews the indications for use of the mathematically directed single-cut osteotomy, describes the mathematical calculations involved in planning, references the required charts to plan the osteotomy, and provides tips and tricks on how to execute the osteotomy intraoperatively.

The surgeon considering this technique should be familiar with the principles of six-axis deformity analysis that define angular and translational deformity using the Cartesian coordinate system in the coronal, sagittal, and axial planes.

## 2. Indications for Use

The incidence of femoral and tibial malunion after a fracture has been reported to be as high as 68% after casting/functional bracing and up to 20% after intramedullary nailing [4,5,6,7,8,9,10,11,12,13,14,15,16]. Inclusive of congenital etiologies, long bone deformities cause pathologic biomechanical forces that can lead to accelerated radiographic and clinical arthritis [17,18,19,20,21]. In general, surgical correction of long bone deformities is recommended in patients with an increased risk of degenerative joint osteoarthritis and for a deformity that causes pain or an unacceptable appearance [22].

Specifically, a mathematically directed single-cut osteotomy is indicated for combined angular and rotational deformity in a diaphyseal or metadiaphyseal long bone segment. Contraindications include (a) significant translation in the coronal, sagittal, and/or axial planes; (b) angular deformity in the coronal and sagittal plane without an axial plane torsional deformity; (c) insufficient bone segment length or integrity to accommodate compression across an extra-articular osteotomy plane; (d) high-magnitude torsional deformity requiring a large axial plane rotational correction; (e) soft tissue contractures or other pathology limiting acute rotation through the osteotomy; and (f) multilevel deformity.

Planning for a mathematically directed single-cut osteotomy does not account for translation. If a preexisting coronal or sagittal translation is present, a resulting Z-type deformity will develop after the correction. Similarly, the osteotomy calculations do not consider preexisting axial translation (shortening or lengthening), although the correction will generate some lengthening of the distal segment, which is predicted using basic trigonometric calculations.

The correction is achieved by rotating the distal segment in the osteotomy plane. This correction will necessarily induce some torsional deformity in the axial plane, the magnitude and direction of which are planned preoperatively. Therefore, some axial plane torsional deformity must be present preoperatively; if no torsion is present, an axial plane deformity will be created.

The goal of a mathematically directed single-cut osteotomy is to correct a deformity while preserving bone and permitting compressive osteosynthesis across a stable osteotomy plane. Very proximal or distal deformities may lack sufficient bone segment length to accommodate the necessary osteotomy plane without violating an adjacent joint surface. Similarly, osseous pathologies, such as cystic changes or active infection, which prevent the safe application of compressive internal fixation, are contradictions to this technique.

The soft tissue must be considered in the planning phase of the mathematically directed single-cut osteotomy. The surrounding soft tissues opposite the apex of the deformity will be stretched during the correction. It may not be possible to fully address deformities that require a large rotational correction if soft tissue contractures, tendon excursion, or tension on neurovascular structures will not permit the required strain. Additionally, the single-cut osteotomy plane is defined by a specific starting point and trajectory. This plane does not consider the appropriate anatomical approach for exposure, saw blade trajectory, or internal fixation. Cicatricial skin that cannot be mobilized should be avoided, especially if it is located at the concavity of the deformity.

The mathematically directed single-cut osteotomy formulas were meant to correct a single-level deformity. Theoretically, concurrent correction of a multi-apical deformity within a single limb segment can be performed with this method. However, the osteotomy plane derivations should be calculated separately for each deformity.

## 3. Geometric Model Definitions

### 3.1. Orthogonal Radiographs

An accurate description of the deformity is required for the useful application of a mathematically directed single-cut osteotomy. Coronal and sagittal deformity analyses involve radiographs that are exactly perpendicular to each other. While these may not be “true” AP and lateral views, the orthogonal orientation allows for a proper trigonometric calculation of the “maximum deformity” and “no deformity” views. These radiographs should be centered at the apex of the deformity and should be reproducible in the operating room when using fluoroscopy. Rotational deformity in the axial plane can be estimated based on the patient’s clinical exam but should be accurately calculated with bilateral CT version studies. Orthogonal two-dimensional simulated radiographic projections, or “ghost reconstruction” views, may also be rendered from CT data for deformity planning.

### 3.2. Six-Axis Deformity Analysis Definitions

A clear understanding of the geometric model and the descriptive variables underpinning a mathematically directed single-cut osteotomy is imperative to proper deformity assessment, osteotomy planning, and corrective execution.

### 3.3. Geometric Model

**Figure 1:**
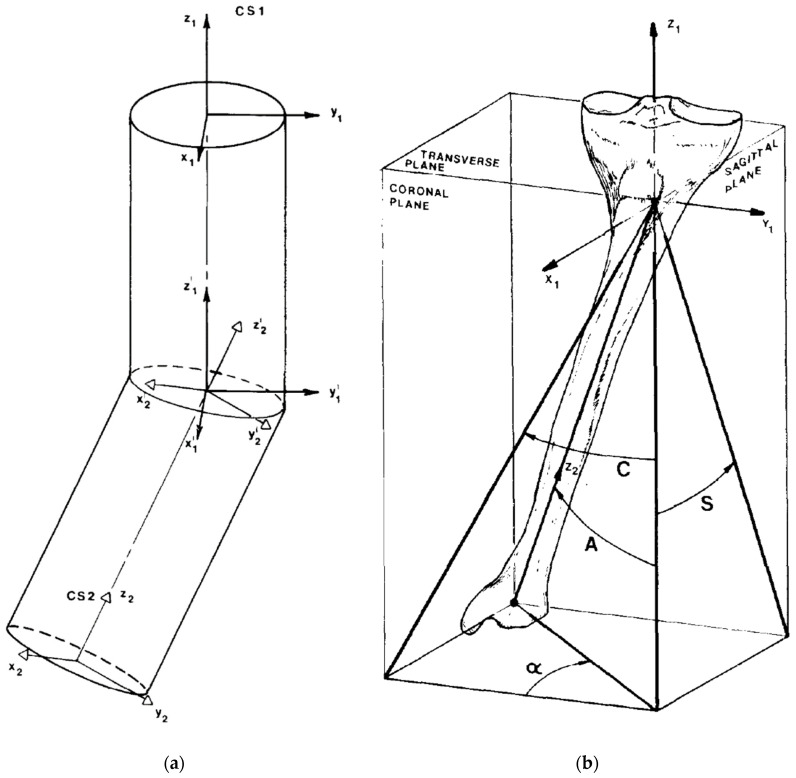
(**a**) The mathematical model describes the deformed bone as two cylinders of equal radii whose axes intersect at the level of deformity to define an elliptical plane. These cylinders are “bent” and rotated relative to each other in space. (**b**) Tibial model with proximal metaphyseal deformity demonstrating projections of the true deformity in the coronal and sagittal views. Angle A is the angulation of the true deformity measured on the maximum deformity view. Angle α, i.e., the orientation angle, is the difference between the coronal plane view and the maximum deformity view [2].

The mathematical model describes the deformed bone as two cylinders of equal radii whose axes intersect at the level of deformity to define an elliptical plane, and which are “bent” or angulated, as well as rotated, relative to each other in space (Figure 1) [2].

As an overview, the parameters C, S, and T are descriptive measurements made by the surgeon based on six-axis deformity analysis definitions. Using these measurements, variables A and α are calculated using simple trigonometric functions. Variables θ and φ are derived using the graphical calculations produced by Sangeorzan et al. and represent the planned osteotomy that is required to simultaneously correct angulation and torsion [1]. Angle β describes the amount of rotation needed to fully correct the deformity intraoperatively and is derived using a trigonometric formula [2]. Below, the input and output variables are referenced.

### 3.4. Descriptive Measurements

C—Coronal angular deformity (degrees)

Angle “C” is the varus or valgus deformity of the distal segment measured on the coronal plane radiograph. By convention, angle C is positive (+) if varus and negative (−) if valgus.

S—Sagittal angular deformity (degrees)

Angle “S” is the flexion or extension deformity of the distal segment measured on the sagittal plane radiograph. Angle S is positive (+) if the deformity is a flexion deformity (apex anterior/procurvatum) and negative (−) if the deformity is in extension (apex posterior/recurvatum).

T—Torsional deformity (degrees)

Angle “T” is the rotation of the proximal segment relative to the distal segment through a plane transverse to the distal segment measured on a clinical exam or a CT version study. By convention, the relative internal rotational deformity is positive (+) and the external rotational deformity is negative (−) for the purposes of the graphical calculations.

### 3.5. Calculated True Deformity

A—True angular deformity (degrees)

Angle “A” is the true deformity angulation between the two cylinders. It is calculated based on angle “C” and angle “S”. It is the total deformity angulation that is represented on the “maximum deformity” view. An orthogonal projection of the true angulation on the “maximum deformity” view would produce a 0° deformity on the “no deformity” view (Figure 1). Angle “A” is calculated using

$$A=\sqrt{C^{2}+S^{2}}$$

α (alpha)—Orientation angle (degrees)

Angle “α” is the orientation of the maximum deformity view relative to the coronal plane view in the axial plane. It is the relative amount of rotation needed to “spin” the coronal view to the maximum deformity view. Angle α will either be positive (+) or negative (−) depending on the sagittal plane deformity direction. Based on the original graphical calculations, α is negative when the deformity is in extension (a negative angle S) regardless of the coronal plane deformity. The +/− direction of angle α is important when calculating the starting point angle φ. Angle α is calculated using

α =arctan(S/C) 

Derived Osteotomy

θ (theta)—Osteotomy angle (degrees)

**Figure 2:**
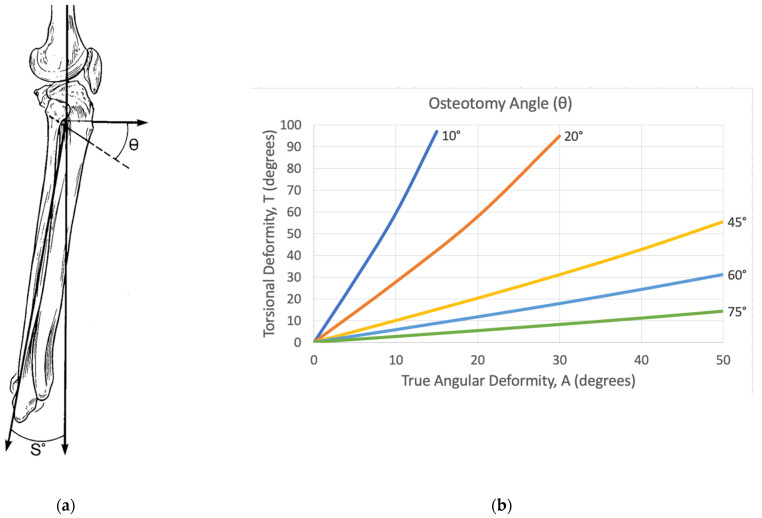
(**a**) Representation of the osteotomy angle theta (θ), as illustrated by Sangeorzan et al. [1]. It describes how steep to make the osteotomy based on the sagittal view. It is referenced from a plane transverse to the long axis of the bone at the level of the deformity. θ is always measured distally. If θ is greater than 90°, the osteotomy plane goes from an ascending to a descending cut. (**b**) Corresponding graph that calculates θ based on the true angular deformity (A) and the rotational deformity T [1].

Angle “θ” is the angulation of the osteotomy viewed from the sagittal plane. It describes how steep to make the osteotomy. It is based on angle A (true angular deformity) and angle T (torsional deformity). If θ is greater than 90°, the osteotomy transitions from an ascending plane to a descending plane. If θ = 0°, the osteotomy would be exactly transverse and all corrections would be rotational corrections. A θ = 90° represents a long bone hemi-section and no rotation would be corrected. Therefore, as the angle θ increases from 0° to 90°, the rotational deformity correction decreases. Angle θ is graphically calculated using the derived Figure 2.

φ (phi)—Starting point angle (degrees)

**Figure 3:**
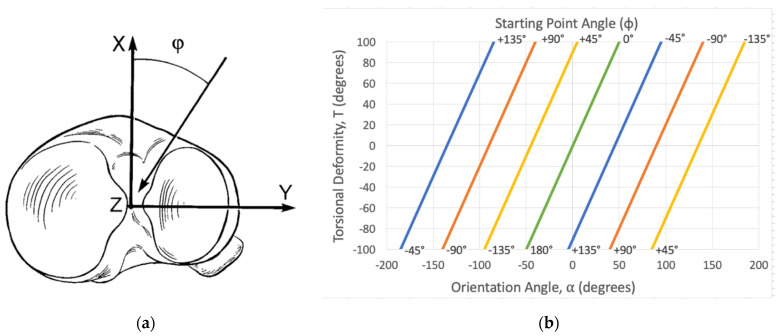
(**a**) Representation of the starting point angle phi (φ), as illustrated by Sangeorzan et al. [1]. It describes how far to rotate the osteotomy plane based on the axial plane. It is referenced from the midline of the long axis. By convention, it is reported as a number less than 180°. A positive number rotates the osteotomy plane laterally and a negative number rotates it medially. (**b**) Corresponding graph that calculates φ based on the orientation angle (α) and the rotational deformity T. For the purposes of the graphical calculations, the relative internal rotational deformity is positive (+) and the relative external rotational deformity is negative (−) [1].

The angle “φ” describes where the osteotomy plane should be in the axial plane. It represents the degree of rotation about the center of the bone to rotate the osteotomy plane. By definition, if the angle φ is positive, the osteotomy is rotated externally (laterally) from the midline. When the angle φ is negative, the osteotomy is rotated internally (medially) from the midline. The angle φ is particularly confusing but is essential to ensure that an angular deformity is not introduced on the “no deformity” view. The natural inclination for the surgeon is to make the osteotomy parallel to the no deformity view; however, this will obligatorily introduce an angular deformity on the no deformity view, even if all other calculations are correct. Angle φ is solved graphically using Figure 3. To ensure the correct use of the graphical calculation for angle φ, a confirmation check should be performed:

$$\varphi \;=\frac{T}{2}-\alpha$$

*This is of critical importance to understand.* Relative to the coronal plane view, the angle φ is equal to half of the torsional deformity minus angle α. By re-referencing the no deformity view (α = 0), the formula simplifies to T/2. Alternatively stated, the angle φ will “move” the osteotomy plane by half of the torsional deformity from the no deformity view. This ensures that an angular deformity is not introduced in the no deformity view.

β (beta)—Angle of rotation (degrees)

Angle “β” is the amount of rotation through the osteotomy plane that is needed to correct the deformity once the osteotomy has been made. Angle β can be calculated pre-operatively using the formula referenced in Sangeorzan et al. [2]. Intraoperatively, this is typically done visually when the osteotomy is straight on all views. Rotational wires can be inserted in both the proximal and distal segments to illustrate the degree of correction.

## 4. Osteotomy Calculations

Using the radiographically measured parameters C and S, the true deformity is calculated. The true deformity is described by angles A and α. Along with the overall torsional angle T” to be corrected, A and α are plotted on the graphs provided by Sangeorzan et al. to output the angles θ and φ [1]. These graphs are based on mathematical models in their original work [2]. The angles θ and φ describe the start point and trajectory of the single-cut corrective osteotomy plane and are used to perform the osteotomy (Figure 2 and Figure 3).

## 5. Performing the Osteotomy

The angles θ and φ define the starting point and trajectory plane of a corrective osteotomy. Fluoroscopic views are referenced that reproduce the orthogonal radiographs used for the deformity analysis. For most long bone corrections, open soft tissue dissection is performed to allow for the visualization and direct bony access. It must be noted that the graphical calculations define the osteotomy and not the exposure. Thoughtful planning should incorporate both the osteotomy and fixation exposures. In general, soft tissue stripping should be minimized to preserve the local blood supply near the osteotomy site.

**Figure 4:**
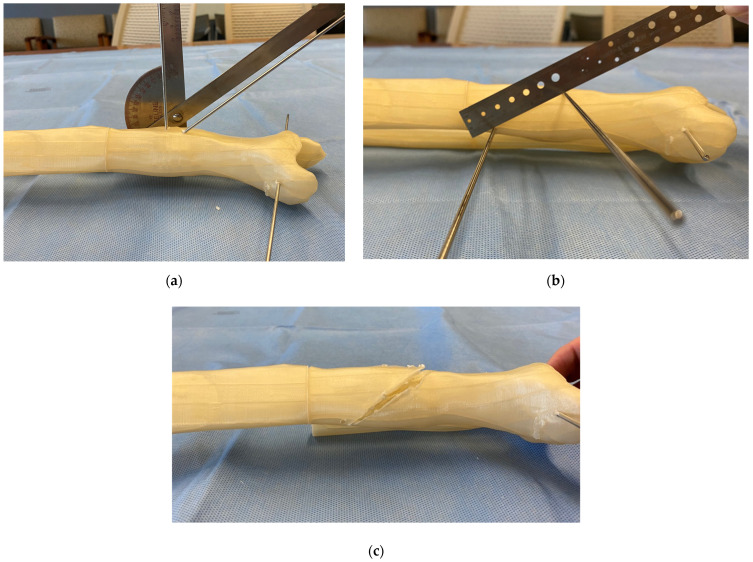
Osteotomy technique demonstrated on a 3D printed model. (**a**) A sterile goniometer or metal triangles are used to precisely execute the osteotomy plane based on the angles θ and φ. (**b**) Multiple K-wires are placed along the planned osteotomy to confirm the correct osteotomy plane and serve as a cutting guide for the saw blade. (**c**) Osteotomy completion and removal of the guide K-wires before rotational correction and fixation.

Several K-wires are placed along the intended osteotomy plane. A sterile goniometer and/or metal triangles are used to accurately place these wires according to the preoperative plan (Figure 4). The K-wires confirm the correct position of the osteotomy, as well as serve as a cutting guide for the saw blade. The osteotomy is typically performed with a rigid saw blade to create a flat cut that is amendable to a rotational reduction. A thinner saw blade minimizes the kerf, thus the amount of bone removed. However, too thin of a blade will cause deflection and an imperfect flat cut.

## 6. Deformity Correction and Osteotomy Fixation

**Figure 5:**
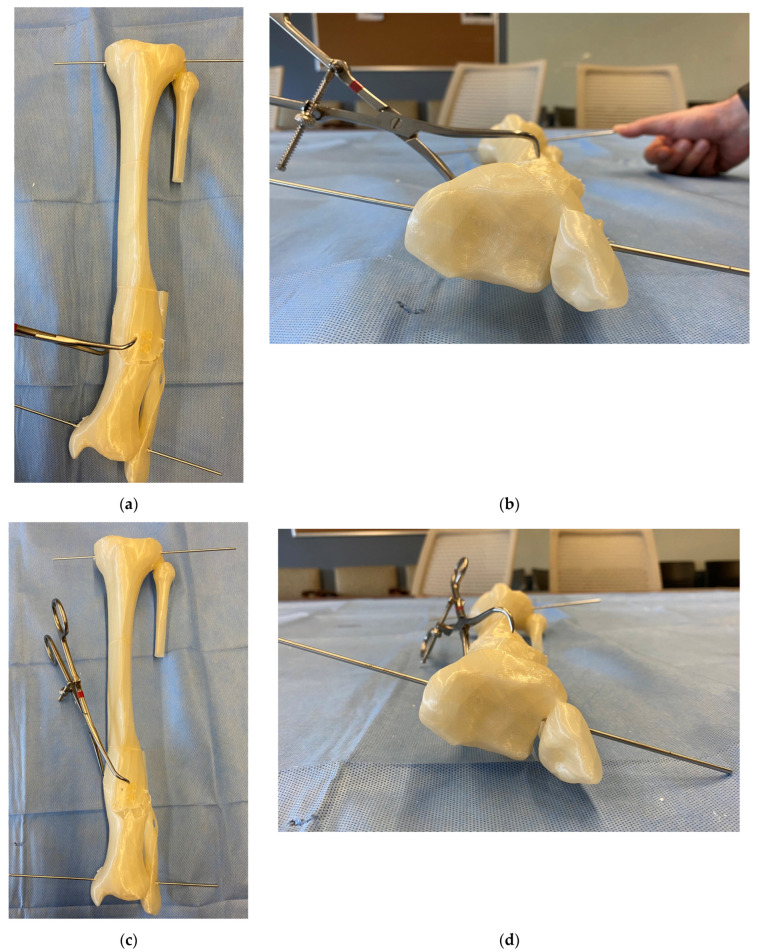
A 3D-printed model before (**a**,**b**) and after (**c**,**d**) a deformity correction with a mathematically directed single-cut osteotomy. It allows for the simultaneous correction of angular and rotational deformity around one osteotomy plane. Note the change in the relative K-wire positions with the correction of the internal rotational deformity on the axial views. This 3D model represents the clinical example in case 2, where the calculation and execution of the mathematically directed osteotomy are described.

Once the osteotomy is complete, an acute reduction is manually performed. No wedge of bone is removed. The calculated angle β represents the degree of rotation required to pivot the distal fragment around the center axis of the osteotomy for a complete correction. Practically, the bone is rotated until it is straight on all intraoperative fluoroscopic views. Reduction clamps or small fragment plates can be used to maintain a provisional reduction (Figure 5).

Because of the single cut without induced translation, the osteotomy is typically amenable to rigid lag screw fixation with non-locking plate neutralization. Alternatively, intramedullary nail fixation may be performed; however, careful assessment of the intramedullary canal must be done, as chronic bone remodeling can preclude a linear canal after a reduction. Insertion of an intramedullary nail into an eccentric canal may induce a translational deformity.

Depending on the direction and magnitude of the correction, concurrent procedures may need to be performed to permit the mobilization of the bone segments. These can include an iliotibial band release, fibular osteotomy, common peroneal nerve release, prophylactic compartment fasciotomies, gastrocnemius–soleus complex lengthening, and stabilization of the proximal and distal tibiofibular joints to prevent subluxation during the correction.

## 7. Supplements and Alternatives

**Figure 6:**
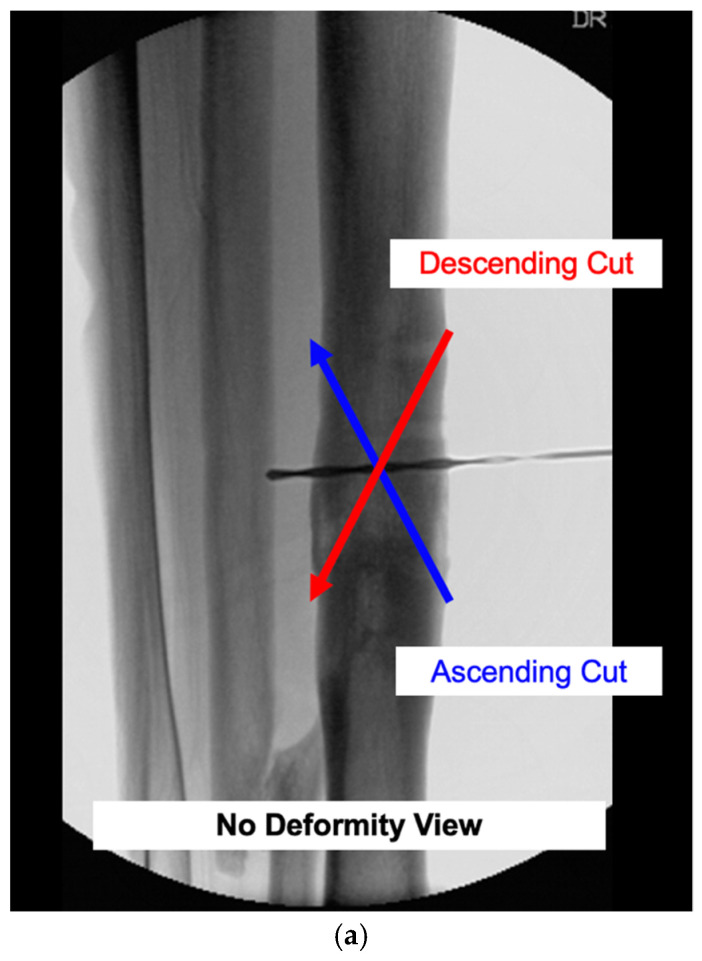

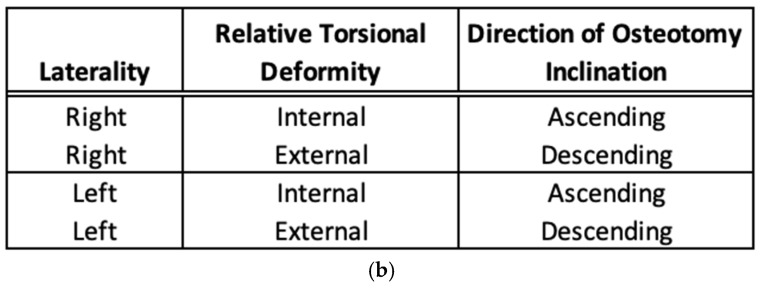
(**a**) The wrong orientation (ascending or descending) of the osteotomy plane will worsen a rotational deformity rather than correct it. To confirm the appropriate osteotomy position, Paccola developed a simple table to determine the appropriate direction of the osteotomy inclination. This is visualized on the no deformity view. The center of rotation of the osteotomy plane is located at the apex of the deformity in the center of the bone. (**b**) Modified from Paccola, this table can be used to verify the appropriate osteotomy plane inclination. It takes into account the limb laterality and the relative torsional deformity to be corrected [23].

An incorrect osteotomy plane orientation (ascending versus descending) will worsen the torsional deformity instead of correcting it. Small changes in torsional correction can be difficult to assess intraoperatively. Because of these concerns, Paccola [23] introduced a simple chart to confirm the appropriate orientation of the osteotomy plane in the no deformity view. He described placing a K-wire or drill bit at the apex of the deformity, then using the table to determine whether the osteotomy should be at an inclination or declination relative to an axis perpendicular to the diaphysis on the no deformity view (Figure 6a). Although this step is mathematically accounted for by the angle θ in Sangeorzan et al.’s paper (θ < 90° is ascending and θ > 90° is descending), Paccola’s table can serve as a simple verification for the osteotomy orientation (Figure 6b).

For the same purposes, Meyer et al. [24] created a physical tool for planning a single-cut corrective osteotomy. This geometric jig consists of two limbs that represent the bony deformity and a plastic disc that automatically orients itself to the osteotomy plane. Their study validated the jig using artificial bone models compared with the charts from the original Sangeorzan papers. While practically useful, this tool is not widely manufactured.

As the correction involves rotation, some lengthening will necessarily occur that can be predicted from the length of the distal segment $Z_{2}$ and the maximum deformity angle A. The amount of limb lengthening can be estimated using the below formula. “Sliding” the bone segments along the osteotomy plane can provide additional length; however, this induces translation and should be reserved for minor length adjustments.

$$∆Z=Z_{2}\left(1-\cos A\right)$$

With advances in computed tomography (CT) and 3D software, patient-specific osteotomy cutting guides have become more widely available. The surgeon may collaborate with a vendor’s engineering team to analyze a deformity, create an osteotomy, fabricate custom cutting jigs, and manufacture patient-specific implants for osteotomy fixation [25]. Although this method is becoming increasingly more popular, it can be costly, and jig application typically requires additional surgical dissection and soft tissue stripping. Additionally, intraoperative navigation systems can assist with deformity correction; however, these may not be available worldwide. Both these adjuvants are relatively new and must be tested for validity and reproducibility. Other alternatives for malunion treatment include acute correction with simple wedge osteotomies, dome osteotomies, gradual correction with an external hexapod frame, or a clamshell osteotomy [26]. Each of these techniques has specific advantages and disadvantages.

## 8. Example Cases

### 8.1. Case 1: Calculating the True Angular Deformity (A) and Orientation Angle (α)

**Figure 7:**
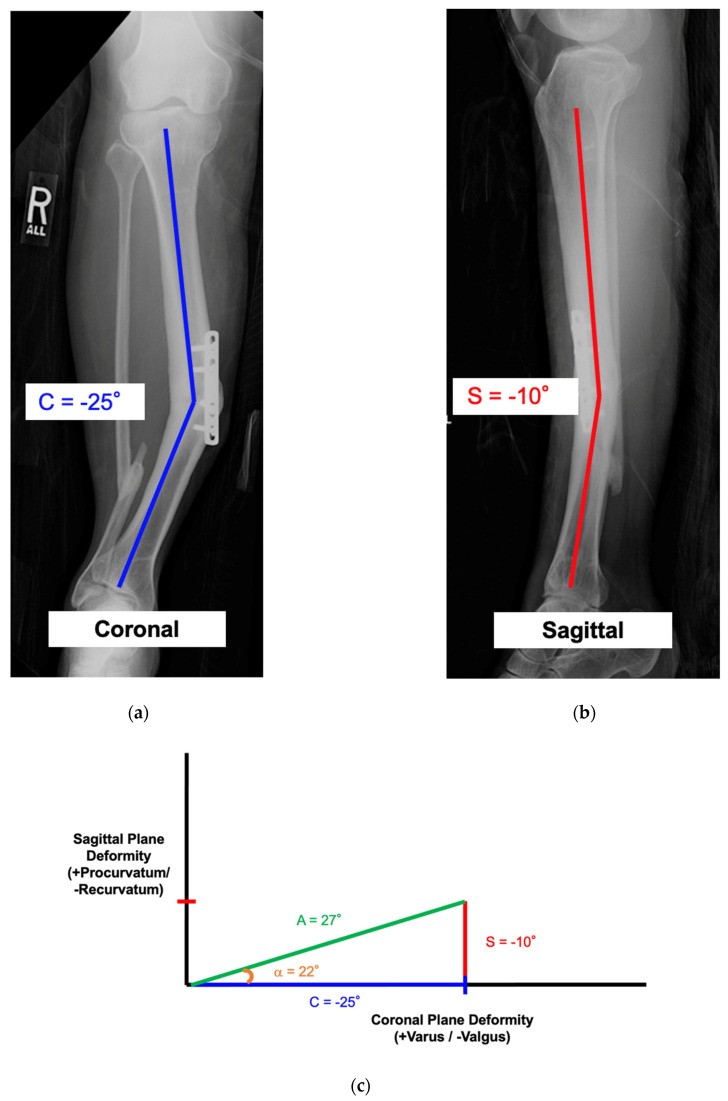

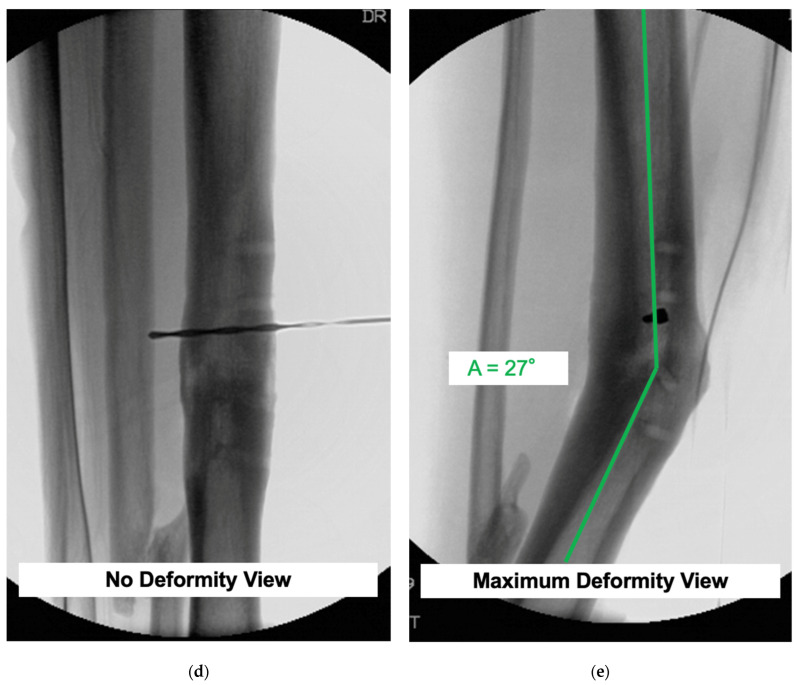
See Case 1 description for details. (**a**–**e**) demonstrate how to calculate the true angular deformity (A) and the orientation angle (α) from coronal and sagittal plane radiographs.

This case demonstrates how to calculate the true angular deformity (A) and the orientation angle (α). The AP and lateral radiographs reveal a −25° valgus and −10° recurvatum deformity of the tibial diaphysis (Figure 7a,b). Measurements for the axial plane deformity (rotation and length) are not required for this step. There is no translational deformity. Using the above trigonometric formulas, the true angular deformity (A) was calculated as 27° and the orientation angle was −22°. A simple triangle diagram could be made for visualization (Figure 7c). The amount needed to rotate the view from the coronal plane view to the “Maximum Deformity View” was −22°. Clinically, this meant that the overall angular deformity was 27° and this maximum angle could be viewed as −22° from the coronal plane view. Figure 7d demonstrates a reference drill bit placed orthogonal to the bone in the “No Deformity View”. Figure 7e demonstrates the true angular deformity (A) in the “Maximum Deformity View”. This view was perpendicular to the “No Deformity View” and the drill bit became coaxial.

### 8.2. Case 2: Calculating the Mathematically Directed Osteotomy

**Figure 8:**
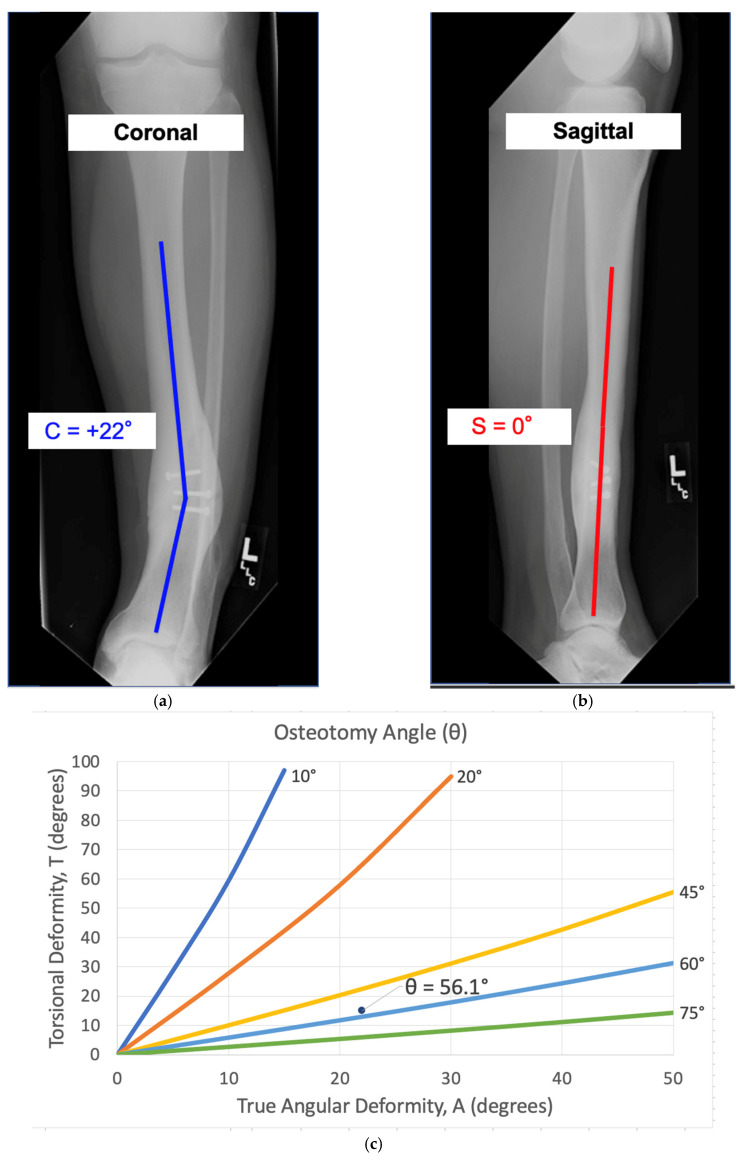

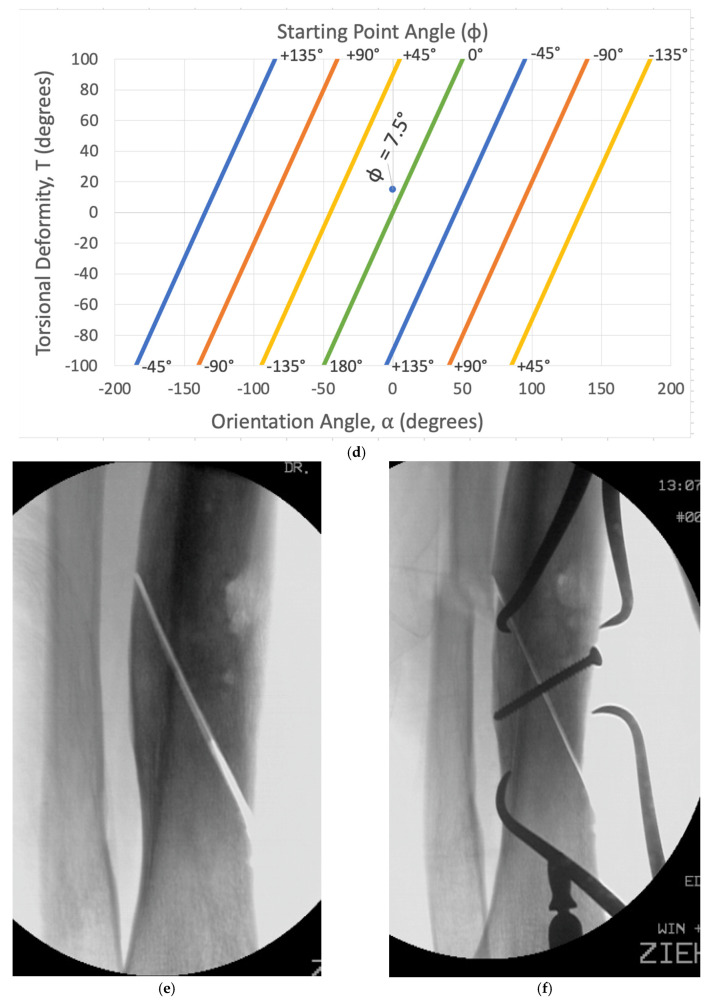

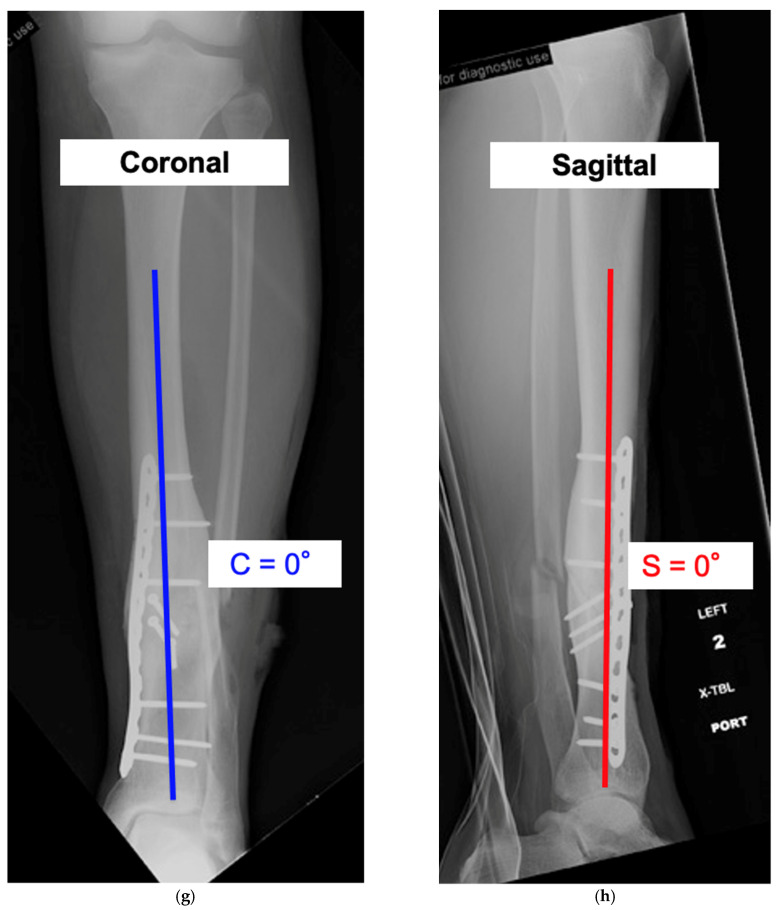
See Case 2 description for details of a tibial malunion. (**a**–**h**) calculate the variables θ (Osteotomy Angle) and φ (Starting Point Angle) to derive the mathematically directed osteotomy. Intraoperative and postoperative radiographs reveal successful correction of the deformity and osteotomy union.

This is a case demonstrating the use of the mathematical charts to calculate the osteotomy angle and orientation. There was a tibial deformity of 22° varus in the coronal plane and a 0° deformity in the sagittal plane (Figure 8a,b). Because there was no deformity in the sagittal plane, the true angular deformity (A) was equal to the deformity in the coronal plane (C) and the orientation angle (α) was 0°. Based on a preoperative CT scan, the patient had a 15° internal rotational deformity (T = +15°). Variables A and T were used to calculate the osteotomy angle (θ = 56°) (Figure 8c). Variables α and T were used to derive the starting point angle (φ = +7.5°) (Figure 8d). To simultaneously correct the 22° angular deformity (A) and 15° rotational deformity (T), the osteotomy plane needed to be tilted 56° ascending (θ) and rotated 7.5° laterally (φ). Figure 8e,f show intraoperative fluoroscopic views of the osteotomy orientation before and after the correction. The osteotomy was made, then the distal segment was rotated until the bone was straight on both the coronal and sagittal views. Lag screw fixation with plate neutralization was done to achieve absolute stability. The final radiographs show the anatomic alignment in the coronal and sagittal planes (Figure 8g,h). In this particular case, there was a simultaneous correction of the coronal plane and internal rotational deformity without induced deformity in the sagittal plane.

## 9. Discussion

Long bone malunions have a reported incidence of up to 68% with casting/functional bracing and as high as 20% with intramedullary nailing [4,5,6,7,8,9,10,11,12,13,14,15,16]. Although malunions can have variable radiographic and functional outcomes, deformity correction is generally recommended for patients with a high risk of degenerative joint osteoarthritis, a painful deformity, or an unacceptable appearance [22]. Deformity correction, including leg length discrepancy, can be treated in several different ways. This includes acute or gradual correction with an external frame, internal fixation, or a combination of both. A mathematically directed osteotomy offers several advantages for the patient with proper malunion characteristics. It requires only one cut, and no wedge of bone is removed. Because the bone surfaces are firmly opposed, direct bone healing can be achieved with compressive fixation. The all-internal hardware avoids specific complications related to external fixation, such as pin site infection, prolonged time in a frame, and the associated psychosocial burden of an external device.

However, this osteotomy is not appropriate for the correction of all malunions. The calculated formula derivations do not account for translation; therefore, malunions with significant translational deformity require a different strategy. An insufficient segment length (periarticular deformity) and poor bone integrity can inhibit direct compressive fixation and is also a relative contra-indication to an MDO. Careful attention to the surrounding soft tissue is required. Acute bony correction in the setting of excessive scar tissue, tight neurovascular structures, or soft tissue contractures may limit the amount of rotational improvement achieved.

Nevertheless, a mathematically directed single-cut osteotomy requires attentive preoperative evaluation and planning, as well as meticulous intraoperative surgical technique.

## 10. Conclusions

A mathematically directed single-cut osteotomy is a precise method for correcting a multiplanar angular and torsional deformity. It is differentiated from the oblique osteotomy as it accounts for induced rotational deformity. Knowledge of its indications for use, osteotomy plane derivations, and tips for surgical execution is paramount in performing a successful correction with a mathematically directed single-cut osteotomy.

## 11. Future Directions

**Figure 9:**
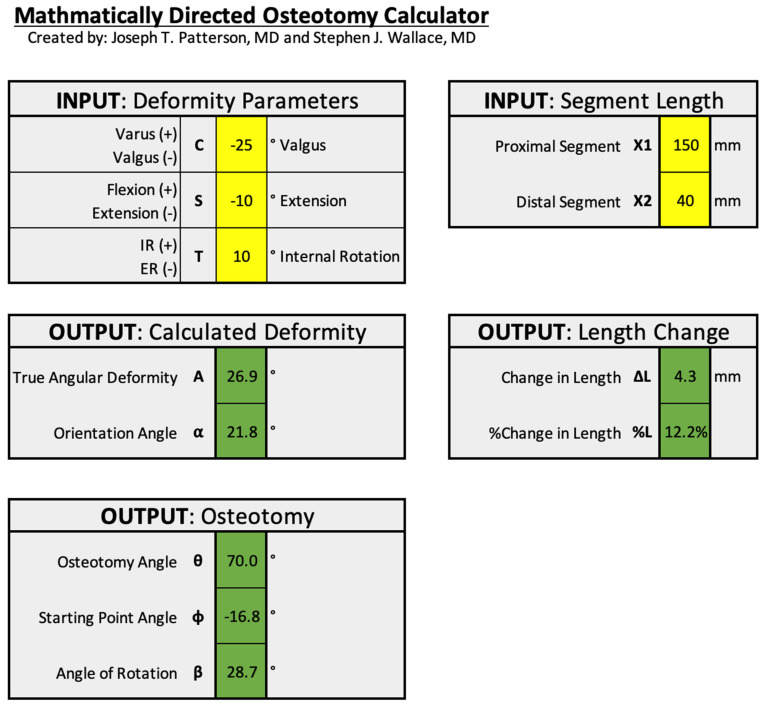
An example of the mathematically derived osteotomy calculator. This tool was developed for ease of use and improved accuracy and precision for MDO planning. Additionally, the program digitally plots the results in a similar format to the original graphs (as seen in cases 1 and 2).

As initially described in 1989, mathematically directed osteotomy planning relied on published graphs for the derivation of the osteotomy plane angles θ and φ. This task can be both tedious and unsettling for those unfamiliar with the process. Furthermore, the calculations are only a close approximation of the true angles due to the limited interval reference lines provided on the graphs. To facilitate planning, the authors have developed a digital MDO calculator (Figure 9). Derived from the original complex mathematical formulas and assumptions, this tool allows for more accurate and precise osteotomy calculations in addition to providing graphical confirmation. For those interested in trialing this calculator, we encourage you to contact the authors.

## Data Availability

Not applicable.
